# The Division of Aging Biology on Basic Research in Health Disparities

**DOI:** 10.1093/geroni/igab046.1402

**Published:** 2021-12-17

**Authors:** Stacy Carrington-Lawrence, Ronald Kohanski

**Affiliations:** 1 Division of Aging Biology/ NIA/ NIH; 2 National Institute on Aging, Bethesda, Maryland, United States

## Abstract

The core mission of the Division of Aging Biology is to explore the molecular and cellular mechanisms of aging. Work supported by the Division is best known for research using laboratory animals, but we have a less well-recognized program of research engaging human participants. With a goal expanding our presence in clinical research and the hope that we can have an impact on health disparities, this talk will provide an overview of how our grantees approach basic mechanistic questions of aging in human communities. In addition, a forward-looking but speculative presentation will be made on ways in which laboratory animals might be used to study health disparities from the perspective of hallmarks of aging.

